# Superconductivity Series in Transition Metal Dichalcogenides by Ionic Gating

**DOI:** 10.1038/srep12534

**Published:** 2015-08-03

**Authors:** Wu Shi, Jianting Ye, Yijin Zhang, Ryuji Suzuki, Masaro Yoshida, Jun Miyazaki, Naoko Inoue, Yu Saito, Yoshihiro Iwasa

**Affiliations:** 1 Quantum-Phase Electronics Center and Department of Applied Physics, The University of Tokyo, 7-3-1 Hongo, Bunkyo-ku, Tokyo, 113-8656, Japan; 2 Zernike Institute for Advanced Materials, University of Groningen, The Netherlands; 3 Center for Emergent Matter Science, RIKEN, Hirosawa 2-1, Wako 351-0198, Japan

## Abstract

Functionalities of two-dimensional (2D) crystals based on semiconducting transition metal dichalcogenides (TMDs) have now stemmed from simple field effect transistors (FETs) to a variety of electronic and opto-valleytronic devices, and even to superconductivity. Among them, superconductivity is the least studied property in TMDs due to methodological difficulty accessing it in different TMD species. Here, we report the systematic study of superconductivity in MoSe_2_, MoTe_2_ and WS_2_ by ionic gating in different regimes. Electrostatic gating using ionic liquid was able to induce superconductivity in MoSe_2_ but not in MoTe_2_ because of inefficient electron accumulation limited by electronic band alignment. Alternative gating using KClO_4_/polyethylene glycol enabled a crossover from surface doping to bulk doping, which induced superconductivities in MoTe_2_ and WS_2_ electrochemically. These new varieties greatly enriched the TMD superconductor families and unveiled critical methodology to expand the capability of ionic gating to other materials.

Semiconducting transition metal dichalcogenides (TMDs) have attracted considerable interest as typical two-dimensional (2D) materials. Atomically flat and chemically stable thin layers of TMDs can be readily obtained via graphene-like mechanical exfoliation^1^ from bulk crystals due to the weak van der Waals interaction-based interlayer bonding^2^. By virtue of their semiconducting nature and defect-free crystal surfaces, thin exfoliated TMD layers are regarded to be ideal channel materials for field-effect transistors (FETs)^3^,^4^,^5^ and TMD-based FETs have been shown to possess remarkable electronic^6^,^7^ and opto-valleytronic properties^8^,^9^,^10^,^11^,^12^,^13^ as well as promising prospects for device applications^14^.

Recent advances in the application of the field effect have been achieved using ionic gating by the formation of electrical double layers (EDLs); an EDL consists of narrow (~1 nm) spatial charge doublets that mimic a capacitor capable of accumulating an ultra-dense sheet of carriers (~10^14^ cm^−2^)^15^. EDL transistors (EDLTs) based on electrostatic ionic gating have proven to be a versatile tool for achieving novel device functionalities^16^,^17^ and inducing new electronic states^18^,^19^,^20^,^21^ at the interface between an ionic medium and a semiconductor channel. The use of EDLTs in TMD research has enabled the investigation of various interesting electronic properties, including ambipolar transport^3^,^5^, electric field control of spin polarization^22^, and circularly polarized electroluminescence^12^. High-density carriers have also bridged the gap to the quantum phases of TMDs through the field effect. The discovery of gate-induced superconductivity in MoS_2_ has revealed enhanced *T*_c_ and a dome-like phase diagram^23^; these features are absent in the chemically doped phase. In light of other available semiconducting TMDs and the effectiveness of electrostatic ionic gating, it is anticipated that this method may be applicable to induce superconductivity in other TMDs.

Following the previous work on MoS_2_^5^,^23^, here, we report a comprehensive study of transport properties and superconductivity in a semiconducting TMD series, specifically 2*H*-type MoSe_2_, MoTe_2_ and WS_2_. Transistor operation and carrier accumulation were significantly influenced by the interfacial energy level alignments of the different TMD materials at the same electrostatic ionic gating using an ionic liquid (IL: DEME-TFSI). Subsequently, the TMDs showed similar gate-induced insulator–metal transitions but did not all reach the superconducting states. In MoSe_2_, new gate-induced superconductivity (GIS) was found with a maximum *T*_c_ of 7.1 K that follows a dome-shaped phase diagram similar to that of MoS_2_^20^,^23^. However, no superconductivity was observed in MoTe_2_ because of the low efficiency of electrostatic electron accumulation that could be achieved in this material using an IL. When the IL was replaced with a KClO_4_/PEG electrolyte, the electron doping could be significantly enhanced through a crossover to an electrochemical regime beyond the electrostatic limit. As a result, superconductivity in 2*H*-MoTe_2_ and WS_2_ were enabled with observed *T*_c_ values of approximately 2.8 K and 8.6 K, respectively. Thus, these results have revealed two series of superconductors in these TMDs. Additionally, this study established new strategies for ionic gating both for electrostatic charge accumulation and electrochemical carrier doping, thereby providing new capability for accessing a wide carrier concentration range and extending superconductivity in other material series.

## Results

### Evolution of electrostatic charge accumulation in MoX_2_ EDLTs

**Figure 1:**
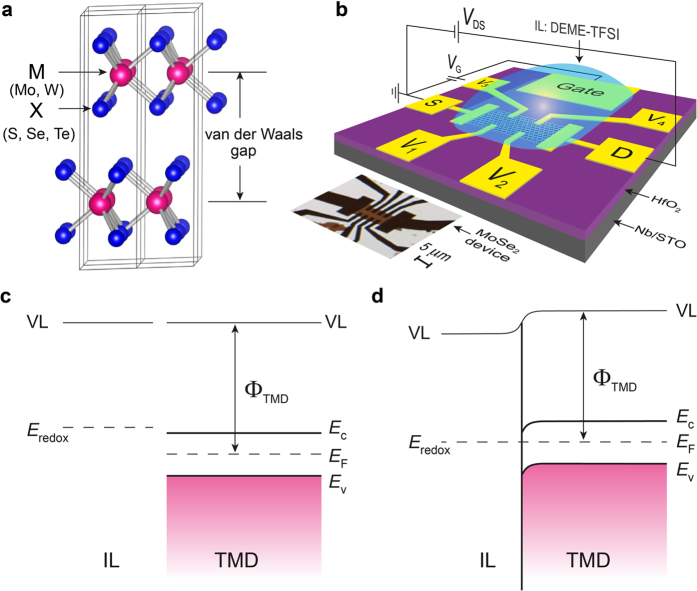
Transition metal dichalcogenide (TMD) EDLT device and schematic diagrams of energy-level alignment at the IL/TMD interface. (**a**) Crystal structure of a 2*H*-type layered transition metal dichalcogenide MX_2_; M = Mo or W and X = S, Se, or Te. (**b**) EDLT device and measurement configuration. The bottom left shows an actual MoSe_2_ nanoflake device with a Hall bar geometry. This figure is drawn by W.S. (**c**) Schematic diagram of the energy levels of an independent ionic liquid and a TMD. (**d**) Schematic diagram of aligned energy levels at the IL/TMD interface. The initial band bending occurs when the IL touches the TMD surface. The vacuum level (VL), redox potential (*E*_redox_), conduction band (*E*_c_), valence band (*E*_v_), Fermi energy (*E*_F_), and work function (*Φ*_TMD_) are defined and labeled in (**c**) and (**d**).

Figure 1a illustrates the crystal structure of 2*H*-type transition metal dichalcogenides, MX_2_ (M = Mo or W; X = S, Se or Te), consisting of two-dimensional covalently bonded X−M−X layers. Due to their weak bonds formed through van der Waals interactions between the layers, thin flakes of MX_2_ can be readily isolated from bulk material via mechanical exfoliation and then fabricated into FET devices (see the Methods section). We used the EDLT structure and employed DEME-TFSI as a gate dielectric; the latter is a widely used IL that has been demonstrated to be capable of accumulating a high density of carriers at the interface even at low bias voltages^15^. Figure 1b presents a schematic diagram of the EDLT configuration and an optical image (bottom left) of a real MX_2_ device (MoSe_2_) prior to the application of the IL. A typical Hall bar geometry was adopted to measure the four-terminal resistance and the Hall carrier density. The thickness of the MoX_2_ flakes in this work ranged from approximately 20 nm to 100 nm as measured by atomic force microscopy (AFM). We confirmed that the 2*H*-type crystal structure was maintained in the thin flakes after transport measurements with IL gating as confirmed by synchrotron microbeam X-ray diffraction experiments (see Fig. S1 and Table S1 in the Supplementary Information).

The gate voltage was applied through a droplet of IL under high vacuum and at a temperature just above the glass transition temperature of DEME-TFSI (i.e., 220 K) to suppress potential chemical reactions between the IL and the film surface^5^. Under these conditions, a TMD thin-flake EDLT can be modeled as a simple contact heterostructure between a semiconductor (i.e., the molybdenum-based TMD) and an electrolyte (i.e., the IL). Following the terminology used for electrolyte/semiconductor interface^24^,^25^,^26^,^27^, Fig. 1c,d present schematic energy-level diagrams before and after making the IL/TMD interface. Here, TMD refers specifically to a 2*H*-type MX_2_ with a finite band gap^28^,^29^. Even before applying a gate voltage, the electric double layer is formed and, in some cases, charge transfer across the interface (electrolysis) takes place until equilibrium is reached, i.e., the redox potential of the electrolyte (*E*_redox_) aligns with the Fermi energy of the semiconductor (*E*_F_)^24^,^25^,^26^,^27^. The space charge layer in the semiconductor has an associated electric field represented by band bending. The work function *Φ*_TMD_ influences the initial charge redistribution and the band bending at the interface before application of gate voltage. Because TMD work functions differ from each other^30^, the initial band bending varies among MoS_2_, MoSe_2_ and MoTe_2_ thin flakes for the same IL. Among these three dichalcogenides, MoTe_2_ exhibits the smallest work function, leading to the weakest initial band bending and the largest carrier injection barrier for electrons. Therefore, a systematic evolution of transistor performance with increasing threshold voltage is expected when the channel material is changed from MoS_2_ to MoTe_2_.

**Figure 2:**
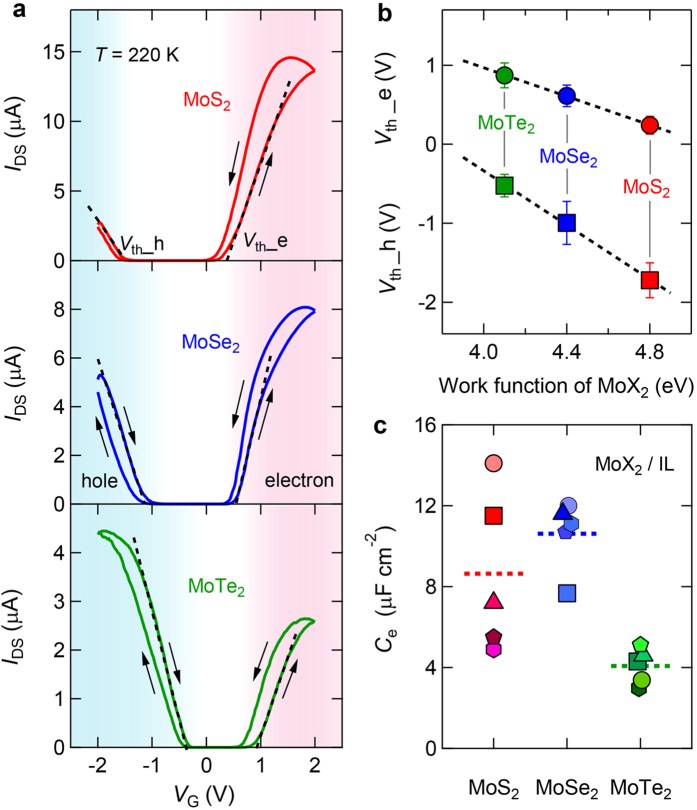
Evolution of ambipolar transfer characteristics and electrostatic charge accumulation in MoX_2_ EDLTs. (**a**) Comparison of typical ambipolar transfer curves of MoS_2_, MoSe_2_ and MoTe_2_ EDLTs measured with *V*_DS_ = 0.1 V at 220 K. *V*_G_ was swept at a constant rate of 20 mV/s through the same IL, DEME-TFSI. The threshold voltages for the electron side (*V*_th__e) and the hole side (*V*_th__h) were determined by linearly extrapolating the *I*_DS_–*V*_G_ curves to zero, as indicated by the black dashed lines. (**b**) Linear correlation between the threshold voltages and the work functions of MoX_2_. The threshold voltages are average values deduced from the transfer curves of 21 MoX_2_ devices with the same IL at 220 K. According to the black dashed lines, a semiconductor with a smaller work function exhibits larger or smaller threshold voltages for electron or hole accumulation, respectively. This can be explained by considering the energy-level alignment at the interface, as shown in Fig. 1d. (**c**) Capacitances for electron accumulation, *C*_e_, deduced from sheet carrier density *n*_2D_–*V*_G_ plots (Fig. S2b) for various MoX_2_ EDLT devices using the same IL, DEME-TFSI. Different symbols represent different devices, and the horizontal short dashed lines correspond to the average values. On average, MoTe_2_ exhibited the lowest EDL capacitance for electron accumulation.

By sweeping the gate voltages at a constant rate of 20 mV/s at 220 K, we measured the transfer curves for the different MoX_2_ (21 devices in total) that were all gated using DEME-TFSI. Figure 2a presents a comparison of the typical transfer curves of MoS_2_, MoSe_2_ and MoTe_2_ EDLTs measured with *V*_DS_ = 0.1 V. All three MoX_2_ transistors displayed ambipolar behavior with systematic properties: within the same bias range, the two extremes of preferences for electron and hole accumulation were dominated by MoS_2_ and MoTe_2_, respectively, whereas MoSe_2_ exhibited a well-balanced ambipolar transistor performance that was most suitable for light-emitting devices^31^. The electron and hole conduction threshold voltages (as indicated by the black dashed lines in Fig. 2a) progressively shifted to higher voltages from MoS_2_ to MoTe_2_. The average values of the threshold voltage *V*_th_ versus the MoX_2_ work function (from ref. 30) are plotted in Fig. 2b, with each data point obtained by averaging 7 devices and the standard deviations shown by the error bars. The electron and hole conduction *V*_th_ values decreased with increasing MoX_2_ work function, consistent with the discussion of the interfacial energy-level alignment presented above. It is well known that in conventional FETs, *V*_th_ is largely governed by the contact effects (work function mismatch between the contact and the semiconductor) and by the deep trap states in the energy gap. However, it is noted that both of these factors take only minor roles in EDLTs^5^,^32^. First, the electrostatic screening (due to the ions close to the metal contact/semiconductor interface) in the liquid can further reduce the width of the Schottky barrier down to values comparable to the electrostatic screening length in the ionic liquid (1–2 nm)^5^,^32^,^33^,^34^. Thus, tunneling-mediated carrier injection is more likely than thermal activation over a Schottky barrier. Second, because of the extremely large EDL capacitance *C*_EDL_ (usually two orders of magnitude larger than the solid gate capacitance *C*_solid_), the threshold voltage shift Δ*V*_th_ (=*N*_trap_/e*C*_EDL_) due to the filling of trap states (*N*_trap_) becomes negligible^32^.

Because our devices were designed to access low-temperature quantum phases, it was critical to quantify the sheet carrier density *n*_2D_, which was therefore unambiguously determined through a Hall effect measurement. To eliminate any temporal change in *n*_2D_ during ionic gating in MoX_2_ EDLTs, all Hall effect measurements were performed at temperatures below the freezing point of ion movement, where the total number of accumulated carriers is fixed. The Hall coefficient *R*_H_ was found to change its sign as a function of the gate voltage *V*_G_ and *n*_2D_ = 1/|*R*_H_*e*| was linearly proportional to the gate bias *V*_G_ in the electrostatic region (Fig. S2). The EDL capacitance can be derived through a linear fit to the *n*_2D_–*V*_G_ plot. We focused on the electron side and compared the capacitances obtained from the *n*_2D_–*V*_G_ plots for 15 different MoX_2_ devices, as shown in Fig. 2c. Even for the same MoX_2_ crystal and IL, the capacitance values were broadly distributed among the devices, possibly because of the existence of different surface states in the individual thin flakes after the fabrication process. However, MoTe_2_ clearly exhibited a smaller average capacitance value (4.1 μF/cm^2^) than MoS_2_ (8.6 μF/cm^2^) or MoSe_2_ (10.6 μF/cm^2^), indicating that MoTe_2_ demonstrated the lowest efficiency in electron accumulation.

### Superconductivity of MoSe_2_ by ionic liquid gating in an electrostatic regime

**Figure 3:**
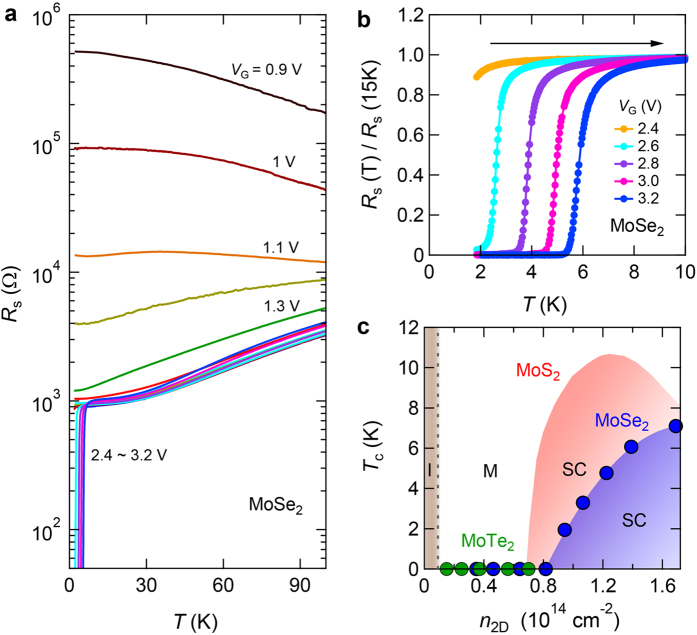
Superconductivity induced by electrostatic IL gating and phase diagram of electron-doped MoSe_2_. (**a**) Temperature dependence of the channel sheet resistance *R*_s_ at various liquid gate voltages for a MoSe_2_ EDLT device using DEME-TFSI as ionic media. (**b**) Normalized channel sheet resistance *R*_s_/*R*_s_ (15 K) of the same MoSe_2_ EDLT device as a function of temperature for various liquid gate voltages from 2.4 V to 3.2 V. (**c**) *T*_c_ versus *n*_2D_ phase diagram of electron-doped MoSe_2_. The MoS_2_ phase diagram (red shade) was taken from ref. 23 for comparison. Here, sheet carrier density *n*_2D_ is obtained from Hall effect measurements and *T*_c_ is defined as the position corresponding to 90% of the total resistance drop. MoTe_2_ data are also presented in (**c**), showing no evidence of superconductivity down to 2 K (*T*_c_ = 0) because of the insufficient maximum carrier density achieved through electrostatic IL gating.

To verify that the electrostatic ionic gating method^23^ described above is applicable for inducing superconductivity in MoSe_2_ and MoTe_2_ thin flake EDLTs, we measured their transport properties down to 2 K by varying *V*_G_ in the positive direction to access electron transport. The results are shown in the plot of the channel sheet resistance *R*_s_ versus *T* in Fig. S3 and the MoSe_2_ data are presented in Fig. 3a. All materials displayed clear insulator–metal transitions with increasing electron density under higher *V*_G_ (Fig. S3). As shown in Fig. 3a, gate-induced superconductivity emerged in MoSe_2_ at *V*_G_ = 2.4 V and developed further with further *V*_G_ increase. However, no superconducting transition was observed in MoTe_2_ up to *V*_G_ = 2.5 V (Fig. S3c), at which point a conductivity maximum was reached. At higher *V*_G_ values, the carrier density was saturated (Fig. S6b) and the mobility decreased, precluding the formation of a more pronounced conducting state. The transport measurements were also conducted for all MoX_2_ devices via hole doping. Similar insulator–metal transitions also occurred as negative *V*_G_ was applied (Fig. S4), but no hole superconductivity was observed down to 2 K for all MoX_2_. Further enhancement of hole density or decrease in temperature is required to achieve hole superconductivity.

Now we consider the properties of the electron-doped superconductor. Figure 3b presents the temperature dependence of the normalized sheet resistance of the MoSe_2_ device at various *V*_G_ (i.e., the device represented in Fig. 3a). It is evident that the superconducting transition can be controlled via electrostatic ionic gating. The *T*_c_ value increased with increasing *V*_G_, reaching a maximum of *T*_c_ = 7.1 K (defined as 90% of the total transition at *V*_G_ = 3.2 V, where *n*_2D_ = 1.69 × 10^14^ cm^−2^). This *T*_c_ value was higher than the maximum previously reported for Sr-doped MoSe_2_ (~5 K)^35^. Based on a precise determination of *n*_2D_ using the Hall effect (Fig. S2), we show the relationship between *T*_c_ and *n*_2D_ for the same MoSe_2_ device in Fig. 3c. In a similar manner to MoS_2_, the superconductivity in MoSe_2_ abruptly emerged above a critical carrier density *n*_0_. Subsequently, *T*_c_ increased with increasing *n*_2D_ until a maximum was reached. This trend was also consistently observed in the *H*_c2_ versus *n*_2D_ phase diagram (Fig. S5). These similarities in the behaviors of MoS_2_ (red shade in Fig. 3c) and MoSe_2_ thus indicated that the dome-shaped phase diagram is a universal feature of gate-induced superconductivity in TMD systems. Figure 3c also shows *n*_2D_ values for multiple MoTe_2_ devices (denoted by green symbols) with *T*_c_ = 0, indicating that no superconducting transitions were observed down to 2 K. We believe that the absence of superconductivity in MoTe_2_ may be due to the low maximum *n*_2D_ of approximately 0.7 × 10^14 ^cm^−2^, which is much smaller than the accumulation on MoS_2_ or MoSe_2_. This difference can be attributed to the fact that MoTe_2_ demonstrated the largest threshold voltage *V*_th_ (bottom panel of Fig. 2a) and the smallest capacitance *C*_e_ (Fig. 2c) during transistor operation in the electrostatic region.

### Superconductivity of MoTe_2_ by electrolyte gating in an intermediate doping regime

**Figure 4:**
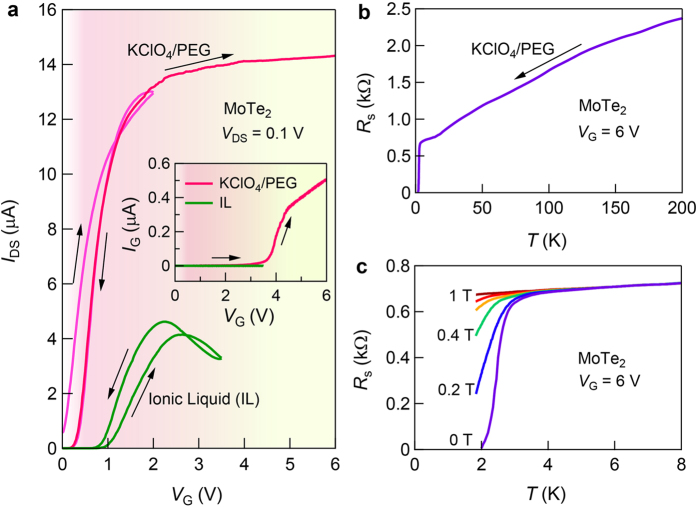
Transistor operation characteristics, *I*_DS_–*V*_G_ and superconductivity in electron-doped MoTe_2_ with KClO_4_/PEG as ionic media. (**a**) Comparison of the transfer curves of 2*H*-MoTe_2_ devices obtained by sweeping the IL gate at 220 K and the KClO_4_/PEG gate at 300 K at constant rate of 20 mV/s with *V*_DS_ = 0.1 V. The inset presents a comparison of the leakage current *I*_G_ as a function of the gate voltage. A reduction in *V*_th__e and a significant enhancement of the ON-state current was observed with the KClO_4_/PEG gate. (**b**) Channel sheet resistance *R*_s_ of the same 2*H*-MoTe_2_ device with the KClO_4_/PEG gate at *V*_G_ = 6 V as a function of temperature. (**c**) Temperature dependence of *R*_s_ for various magnetic fields from 0 T to 1 T. The superconducting transition temperature *T*_c_, defined as the temperature corresponding to 90% of the normal-state resistance at 1 T, is 2.8 K at *V*_G_ = 6 V.

To induce superconductivity in MoTe_2_, it is necessary to enhance the carrier density through more efficient transistor operation. The threshold voltage can be reduced by choosing ILs with smaller work functions. However, this achieved only a moderate enhancement in the carrier density because of the slight increase in capacitance and a significant *n*_2D_ saturation observed with increasing *V*_G_ (Fig. S6). We then found that the limitations on carrier accumulation could be surmounted by replacing the organic IL with the KClO_4_/PEG electrolyte. Figure 4a compares the channel current values *I*_DS_ for MoTe_2_ devices fabricated using the two ionic media: the IL (DEME-TFSI) and the KClO_4_/PEG electrolyte. For IL gating, an increase in *I*_DS_ was initiated at a large threshold voltage of approximately 1 V and was no longer observed when *V*_G_ exceeded 2.5 V, indicating a limit on the formation of a more conductive channel. This peak behavior of *I*_DS_ versus *V*_G_ was associated with *n*_2D_ saturation and was confirmed in multiple devices, implying that the observed behavior was not related to device degradation. For KClO_4_/PEG electrolyte gating, electrostatic electron accumulation occurred at a much smaller threshold voltage and induced a rapid increase of *I*_DS_, reaching a high conducting state at *V*_G_ approximately 3.5 V. The conducting state was then sustained as *V*_G_ was continuously increased up to 6 V. This gave rise to the observation of good metallic conduction followed by a superconducting transition at low temperatures, as shown in Fig. 4b. The magnetic field dependence of the *R*_s_−T curves (Fig. 4c) further confirmed the existence of superconductivity at *T*_c_ = 2.8 K (defined as the temperature corresponding to 90% of the normal-state resistance at *H* = 1 T). Since superconductivity in MoTe_2_ has never been reported, the result in Fig. 4 marks the discovery of a new superconductor in the 2*H*-MoX_2_ family.

Limited by the much lower ionic mobility in an electrolyte compared with an IL, we applied a *V*_G_ at 300 K for the KClO_4_/PEG electrolyte; at this temperature, the ions were sufficiently mobile. At such a high temperature, an increase in the leakage current associated with the saturation of *I*_DS_ was observed above *V*_G_ = 3.5 V (as shown in the inset of Fig. 4a), indicating that electrolysis of PEG or potassium intercalation may have occurred. At 20 K, *n*_2D_ reached 0.9 × 10^14^ cm^−2^, only slightly higher than the maximum observed for IL gating. We define this voltage range for nearly saturated *I*_DS_ as an intermediate regime beyond the electrostatic limit as it could be followed by an electrochemical regime at a higher *V*_G_ where potassium intercalation may occur more effectively into the entire flake and eventually become dominant.

### Superconductivity of WS_2_ by electrolyte gating in an electrochemical regime

**Figure 5:**
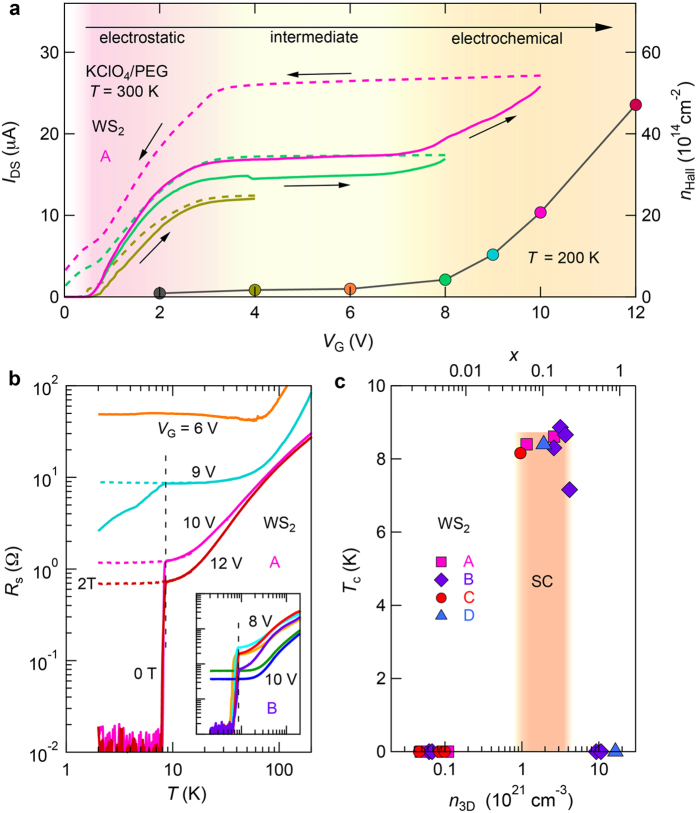
*I*_DS_–*V*_G_ characteristics and superconductivity in WS_2_ induced by electrochemical doping with KClO_4_/PEG as ionic media. (**a**) Channel current *I*_DS_ (left axis) versus *V*_G_ characteristics of the WS_2_ device A obtained by sweeping the KClO_4_/PEG gate at 300 K at constant rate of 20 mV/s with *V*_DS_ = 0.1 V. The solid lines indicate the forward *V*_G_ scan of *I*_DS_, followed by immediate cooling of the device for low temperature transport measurements fixed at each maximum *V*_G_. The dashed lines were obtained via a backward scan of *V*_G_ after the device was warmed up to 300 K. Right axis shows carrier density *n*_Hall_ (denoted by circle symbols) plotted as a function of *V*_G_ measured at 200 K. (**b**) Temperature dependence of *R*_s_ in log scale for the same WS_2_ device A at various *V*_G_ from 6 V to 12 V. The solid and dashed curves represent the data obtained for magnetic fields at 0 T and 2 T, respectively. The inset shows *R*_s_ versus *T* in the same log scale for another WS_2_ device B at various *V*_G_ from 8 V to 10 V. The vertical dashed line indicates that the same onset *T*_c_ was observed for different *V*_G_, implying the existence of a line-phase K_x_WS_2_ compound. (**c**) Phase diagram of electron-doped WS_2_ as a function of bulk carrier density *n*_3D_ (bottom horizontal axis) and doping concentration *x* (upper horizontal axis). The superconducting transition temperature *T*_c_ is defined as the position corresponding to 90% of the total resistance drop. *n*_3D_ and *x* is calculated from Hall effect and the thickness of the flake by assuming that the whole thin flake is uniformly doped. Different filled symbols represent different devices and the shaded area defines a complete superconducting (SC) region reaching “zero resistance”.

WS_2_ is another member of semiconducting TMDs, where bulk superconductivity was known to occur at 3.5 K by conventional chemical doping of Sr^35^. Recently, ionic gating using IL was found to induce superconductivity at 4 K^36^. To confirm the different doping mechanism using the KClO_4_/PEG electrolyte as ionic media and gain access deeper into the electrochemical regime in WS_2_, we further increased our voltage bias up to 12 V and systematically studied the evolution of carrier accumulation in a WS_2_ device labeled as A. Figure 5a presents the channel current values *I*_DS_ as a function of *V*_G_ measured at 300 K (see the complete data in Fig. S7). We can see that *I*_DS_ went through the similar electrostatic and intermediate doping regimes reaching a limited value similar to that of MoTe_2_ described above; however, here, the limited *I*_DS_ (approximately 17 μA at *V*_G_ = 6 V) corresponded to a metallic state that failed to access the superconducting region. This limit was clearly broken when *V*_G_ exceeded approximately 7 V, where a second rapid upturn of *I*_DS_ strongly indicated an effective electrochemical doping process, *i.e*., ion intercalation under high bias. This was supported by the distinctive large hysteresis (magenta dashed line in Fig. 5a) manifesting the intercalation and de-intercalation in a *V*_G_ scan cycle. Because the device nearly recovered its pristine state after *V*_G_ was restored back to zero, the low temperature transport can be measured after the *in situ* change of *V*_G_ in a similar way as for the measurements in the electrostatic regime using ionic liquids. As shown in Fig. 5a, we also measured the carrier density *n*_Hall_ by the Hall effect at 200 K (also see Fig. S8). Here, *n*_Hall_ corresponds to the sheet carrier density *n*_2D_ in the electrostatic regime, whereas beyond the electrostatic regime, *n*_Hall_ corresponds to the projected carrier density, which is essentially the bulk density *n*_3D_ multiplied by the thickness of the doped sample. Examination of the *n*_2D_ values (as shown in Fig. 5a) measured by the Hall effect at 200 K (denoted by circle symbols) showed that in the intermediate region, *n*_Hall_ increased very slowly until reaching an upper limit *V*_G_ of approximately 7 V; this is followed by a rapid *n*_Hall_ increase reaching *n*_Hall_ = 4.7 × 10^15^ cm^−2^ at *V*_G_ = 12 V, more than one order of magnitude higher than the maximum value obtained in the electrostatic region. Accordingly, the capacitance deduced from the *n*_Hall_–*V*_G_ data in the electrochemical region was much higher, achieving up to 200 μF/cm^2^, providing strong evidence that the carrier doping reaches far beyond the level achieved in the electrostatic regime.

We then focused on the transport properties of the electrochemically doped WS_2_. Figure 5b presents the results for *R*_S_ as a function of temperature on the log scale for device A at various *V*_G_ from 6 V to 12 V. We observed good metallic conduction followed by a superconducting transition at the same onset transition temperature (the dashed vertical line in Fig. 5b) for *V*_G_ at 9 V, 10 V and 12 V. The transition can be completely destroyed by applying a magnetic field of 2 T as indicated by the dashed curves in Fig. 5b, providing strong evidence of superconductivity. Unlike the gate modulation of *T*_c_ observed in IL-gated MoSe_2_ (Fig. 3b), here, the onset *T*_c_ value did not change with *V*_G_. One possible explanation is that the superconducting phase is a line-phase compound in terms of K concentration. Potassium ions started to intercalate between the WS_2_ layers when *V*_G_ exceeded a critical value (~7 V for device A). Then, a particular K_x_WS_2_ line-phase compound that exhibited a superconducting transition with a fixed *T*_c_ was formed at approximately 9 V. In this initial state where superconductivity appeared, the sample consists of a mixture of nonsuperconducting phase with lower x and the superconducting phase. As *V*_G_ further increased, the superconducting phase gradually grew and finally occupied a substantial portion of the whole channel, reaching the “zero resistance” state (*R*_s_ < 0.02 Ω, limited by our measurement resolution). This is also supported by the gradual decrease of the normal state resistance with increasing *V*_G_ (Fig. 5b).

Similar superconducting transitions were also observed in other devices. The inset in Fig. 5b presents *R*_s_ versus *T* curves in the same log scale for another WS_2_ sample (device B). Superconducting transitions were well developed down to “zero resistance” for *V*_G_ = 8 V. When intercalation further proceeded, an overdoped phase started to grow, forming a mixture of the superconducting phase and the overdoped non-superconducting phase. Finally, zero-resistance disappeared since an overdoped metallic state became dominant at *V*_G_ = 10 V, where a high *n*_Hall_ of approximately 2.1 × 10^16^ cm^−2^ was achieved. With the ability of reaching such a high carrier density, this gate-controllable intercalation process is also applicable to metallic TMD system^37^. The critical *V*_G_ varies from sample to sample due to the differences in sample thicknesses and device configurations even following the same gating procedure. Here, *n*_Hall_ is important to quantify the doping level of the flake. Assuming that the whole flake was uniformly doped, the bulk carrier density *n*_3D_ and the doping concentration *x* can be deduced from the Hall effect and the thickness of the thin flake. Figure 5c shows the phase diagram for *T*_c_ versus *n*_3D_ (bottom horizontal axis) and doping concentration *x* (upper horizontal axis) collected from four different devices with superconducting transitions reaching “zero resistance”, where *T*_c_ was defined as the temperature corresponding to 90% of the total transition. The similar *T*_c_ values (approximately 8.6 K) within the shaded area support the existence of a line-phase superconducting phase in K_x_WS_2_ between *x* = 0.05 and 0.2. This is consistent with the conventional staging effect frequently observed in the intercalated states of layered systems^38^. The chemical composition *x* of the superconducting phase should be accurately determined by Raman spectroscopy and X-ray diffraction. When potassium is intercalated beyond the optimal range, superconductivity finally disappeared (*T*_c_ = 0), implying a possible phase change to the overdoped non-superconducting compound. Fig. 5b shows that the electrochemically doped WS_2_ exhibited an abrupt superconducting transition at *T*_c_. This result contrasts strikingly with the electrostatically gated MoSe_2_, for which we observed rather broad superconducting transitions that can be interpreted as a fluctuation phenomenon of the 2D nature of superconductivity^39^. Similar feature is also observed in other electric field induced superconductivity, for instance, in SrTiO_3_^40^. The sharp resistance drop in the K intercalated WS_2_, on the other hand, indicates that this system behaves as a three-dimensional (3D) or at least anisotropic 3D superconductor.

## Discussion

In this study, we report the discovery of superconductivity in 2*H*-type MoSe_2_, MoTe_2_ and WS_2_ induced by a crossover from electrostatic to electrochemical doping as an exploratory tool to access wide and controllable doping regimes. Following the established electrostatic doping, we discovered the presence of previously unknown gate-induced superconductivity in MoSe_2_ but failed to find it in MoTe_2_ because of the lower efficiency of electron accumulation in the latter. By replacing the IL with a KClO_4_/PEG electrolyte, we accessed the adjacent intermediate doping regime and further electrochemical doping regime beyond the electrostatic limit, where superconductivity was successfully discovered for MoTe_2_ and WS_2_. To the best of our knowledge, these two materials are new, unprecedented superconductors. The discovery of a superconductor series in Mo and W chalcogenides proves that superconductivity is a common property for semiconducting TMDs. Additionally, this study provides new strategies and guidelines for the ionic gating technique: optimization of the energy level alignment at the interface for electrostatic carrier accumulation and the use of polymer electrolyte and high gate bias to induce electrochemical doping beyond the electrostatic limit. Combination of these two doping regimes can significantly broaden the applicability of ionic gating, making it a versatile tool for the study of a wide variety of materials. Thus, this study improves the versatility and effectiveness of the ionic gating method, which may play an essential role, in combination with conventional chemical doping, in the discovery of new superconductors.

## Methods

### Crystal growth

MoS_2_ single crystals were obtained commercially (SPI Supplies). MoSe_2_ single crystals were grown via a chemical vapor transport (CVT) technique^41^,^42^. Initially, Mo and Se powders were sealed in a quartz tube. The mixture was transported by iodine gas for 14 days before the crystals were grown in a two-zone furnace with a horizontal temperature gradient that was established by maintaining the higher-temperature side (*T*_H_) at 1050 °C and the lower-temperature side (*T*_L_) at 950 °C. MoTe_2_ and WS_2_ single crystals were grown using the same method, with chlorine gas as the transporting agent and with a gradient established by maintaining *T*_H_/*T*_L_ at 800 °C/750 °C for MoTe_2_ and *T*_H_/*T*_L_ at 1000 °C/700 °C for WS_2_.

### Device fabrication

Multilayered thin flakes of MX_2_ were cleaved from the bulk, as-grown single crystals using the scotch tape method. Subsequently, the flakes were transferred to Nb-doped SrTiO_3_ substrates covered with a 30 nm HfO_2_ layer (grown via atomic layer deposition) and SiO_2_ (300 nm)/Si substrates. We chose atomically flat thin flakes with thicknesses from 20 nm to 100 nm (by using an optical microscope)^5^, which were then patterned into a Hall bar configuration via conventional microfabrication techniques. Then, electrodes were prepared by evaporating layers of 5 nm Ti/60 nm Au. A rectangular channel area was defined using a 100 nm SiO_2_ negative mask or a thick electron beam resist (approximately 400 nm ZEP 520A) layer^5^,^19^,^23^. The ionic medium, i.e., the ionic liquid N,N-diethyl-N-methyl-N-(2-methoxyethyl) ammonium bis(trifluoromethanesulfonyl) imide (DEME-TFSI) or the KClO_4_/PEG polymer electrolyte, was applied to the top of the thin flake and the side gate electrode for ionic gating. The device configuration is illustrated in Fig. 1b. The polymer electrolyte was prepared by dissolving KClO_4_ in polyethylene glycol (PEG; *M*_w_ = 600) with a [K]:[O] ratio of 1:20. The solution was liquid at 300 K and underwent a glass transition below approximately 250 K.

### Measurement details

All transport measurements were performed using a Quantum Design Physical Property Measurement System (Quantum Design, Inc.) in high-vacuum mode (10^−5^ Torr). To reduce the likelihood of chemical reactions between the IL and the thin flakes, the transfer curves were acquired using a constant source–drain bias voltage of 0.1 V at a gate-voltage sweep rate of 20 mV/s at 220 K^5^,^15^. The temperature dependences of the resistance data collected at various gate voltages were measured using a previously established process^19^, *i.e*., first performing ionic gating at 220 K, followed by a cooling period under the applied voltage and subsequent warming to 220 K for gating to another *V*_G_. When the KClO_4_/PEG electrolyte was used, *V*_G_ was applied at 300 K.

## Additional Information

**How to cite this article**: Shi, W. *et al*. Superconductivity Series in Transition Metal Dichalcogenides by Ionic Gating. *Sci. Rep*. **5**, 12534; doi: 10.1038/srep12534 (2015).

## Supplementary Material

Supplementary Information
