# Supplementary material for: Superconductivity Series in Transition Metal Dichalcogenides by Ionic Gating
Source: Sci Rep. 2015 Aug 3;5:12534. doi: 10.1038/srep12534 (PMC4522664; doi:10.1038/srep12534)
Supplement: Supplementary Information [file srep12534-s1.pdf]

Superconductivity Series of Transition Metal Dichalcogenides by Ionic Gating

Wu Shi[1], Jianting Ye[1,2], Yijin Zhang[1], Ryuji Suzuki[1], Masaro Yoshida[1], Jun Miyazaki[1], Naoko Inoue[1], Yu Saito[1], and Yoshihiro Iwasa[1,3]

[1] *Quantum-Phase Electronics Center and Department of Applied Physics, The University of Tokyo, 7-3-1 Hongo, Bunkyo-ku, Tokyo, 113-8656, Japan*

[2] *Zernike Institute for Advanced Materials, University of Groningen, The Netherlands*

[3] *Center for Emergent Matter Science, RIKEN, Hirosawa 2-1, Wako 351-0198, Japan*

Supplementary Information

1. Characterization of MX_2 single crystals and cleaved thin flakes

We prepared MX_2 single crystals as described in the Methods section; the crystals were characterized via powder X-ray diffraction. The measured lattice constants are listed in Table S1 and were consistent with values reported in the literature[1, 2]. Thin flakes were obtained via mechanical cleavage from bulk single crystals and were fabricated into devices with Hall bar geometries on HfO_2 (30 nm)/Nb-doped $SrTiO_3$ substrates for transport measurements. Conventional SiO_2 (300 nm)/Si substrates were also used in some devices. To confirm the chemical stability of the MoX_2 thin flakes during the ionic liquid (IL) gating process, we performed AFM and thin-film X-ray characterization after the transport measurements. Figure S1a presents an AFM image of an $MoSe_2$ thin-flake device on an SiO_2/Si substrate, which exhibited gate-induced superconductivity in low-temperature measurements and was subsequently cleaned by removing the IL after it had warmed up. As seen from Fig. S1a, the flake thickness was approximately 59 nm and the root mean square of the roughness was approximately 0.8 nm, indicating that a high surface quality was maintained after IL gating. Figure S1b presents the synchrotron microbeam X-ray diffraction measurements of two other devices that displayed superconductivity. The experiments were conducted at the BL13XU beamline at SPring-8. The synchrotron X-ray energy was 12.4 keV, and the beam radius was 0.4 μm, which was sufficiently small to target the channel area of the nanoflake that was instrumented with electrodes. The position of the channel area was adjusted to the focal point of the microbeam X-ray by monitoring the fluorescent X-rays emitted by the gold electrode. The *c*-axis lattice constant was deduced to be 6.4784(2) Å from the (008) plane diffraction peak through fitting with a Gaussian function; this result was consistent with

the value for bulk single crystals (see Table S1), suggesting that the IL gating effect was free of chemical intercalation[3].

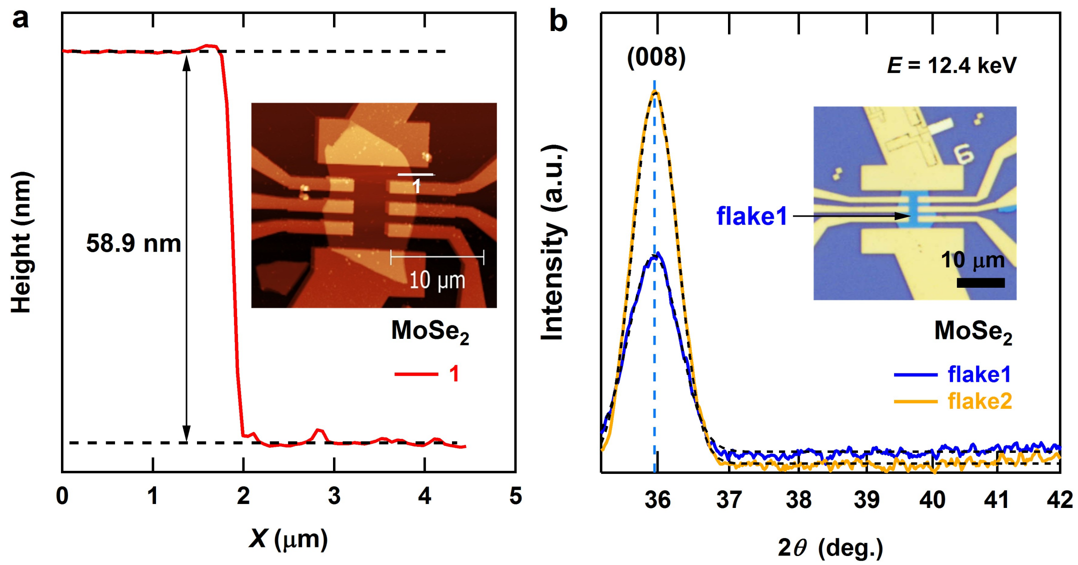

Supplementary Figure S1 | Characterization of cleaved $MoSe_2$ thin flakes. (**a**) Height profile and AFM image of a 2*H*-$MoSe_2$ thin-flake device with a Hall bar geometry. The device was cleaned by removing the IL on top after the low-temperature transport measurements were completed. The thickness of the flake was measured from the height profile to be 58.9 nm. This figure is drawn by W.S.. (**b**) Synchrotron microbeam X-ray diffraction results of two other $MoSe_2$ thin-flake devices on SiO_2/Si substrates. The inset shows an optical image of flake 1. The synchrotron X-ray energy was 12.4 keV. The black dashed curves represent fits with a Gaussian function. A Bragg peak associated with (008) plane scattering is clearly observed at an angle of 35.96 degrees, corresponding to a *c*-axis lattice constant of 6.4782(2) Å.

Supplementary Table S1. Properties of 2*H*-MX_2 single crystals

		2*H*-MoS_2	2*H*-$MoSe_2$	2*H*-$MoTe_2$	2*H*-WS_2
Lattice constant [Å]*	*a*	3.1578(1)	3.2907(1)	3.5161(3)	3.1606(1)
	c	6.1343(5)	6.4656(1)	6.9607(5)	6.1756(2)
Lattice constant [Å][1, 2, 4]	*a*	3.160	3.288	3.517	3.153
	c	6.147	6.460	6.981	6.162
Band gap energy [eV][5]	*Bulk*	1.29	1.1	1.0	1.35
	Monolayer	1.89	1.58	1.23	2.05
Work function [eV][6, 7]	*Bulk*	4.8	4.4	4.1	4.2

* from our experimental results by X-ray diffraction. The Cu source was used for MoS_2, $MoSe_2$ and WS_2 while the Mo source was used for $MoTe_2$.

2. Hall effect measurements and transport properties of IL-gated $MoSe_2$

We carried out Hall effect measurements under magnetic field B up to 9 T in order to confirm the carrier polarity and determine the sheet carrier density n. Figure S2a shows the Hall resistance R_{xy} as a function of magnetic field B measured at 20 K for different gate voltages from the same $MoSe_2$ thin flake EDLT device as Fig. 3. The black solid lines are the linear fit to the curves. The slope $R_H = dR_{xy}/dB$ clearly changes its sign when V_G switches from positive to negative, indicating the change of carrier polarity from electron to hole in the channel. It clearly proves that ambipolar operation is realized in the EDLT device by switching V_G.

Figures S2b-d display the absolute sheet carrier density $n_{2D} = 1/|R_H e|$, the Hall mobility μ_H and the sheet conductivity σ_s, respectively, of the same $MoSe_2$ device at 20 K as a function of the gate voltage. The Hall mobility was derived from $\mu_H = \sigma_s/|n_{2D}e|$, and the sheet conductivity σ_s was obtained via a four-terminal resistance measurement. The curves presented with solid and open symbols correspond to electron and hole conduction, respectively. The n_{2D}–V_G plot reveals linear dependences (dashed lines) in both cases in Fig. S2b, indicating an electrostatic charging process. The EDL capacitance was calculated through a linear fit to the n_{2D}–V_G data, where $C = \mathrm{d}n_{2D}e/\mathrm{d}V_G$. The electron and hole capacitance values were found to be C_e = 11.1 μF/cm^2 and C_h = 10.5 μF/cm^2, respectively. As seen in Fig. S2c, the Hall mobility decreased with increasing V_G after reaching a peak value. As a consequence, the conductivity did not monotonically increase with increasing V_G; instead, it exhibited a pronounced peak, as displayed in Fig. S2d. It is noted that the considerable decrease in mobility with increasing V_G that is seen in Fig. S2c has also been observed in $KTaO_3$ EDLT and rubrene EDLT systems[8-10], possibly as a result of enhanced surface scattering under high gate bias[8] and strong binding interactions between anions and holes at the rubrene/liquid interface that localized the charge carriers[9, 10], respectively. The decrease in mobility with increasing V_G in MoX_2 can also likely be understood to be a result of these mechanisms.

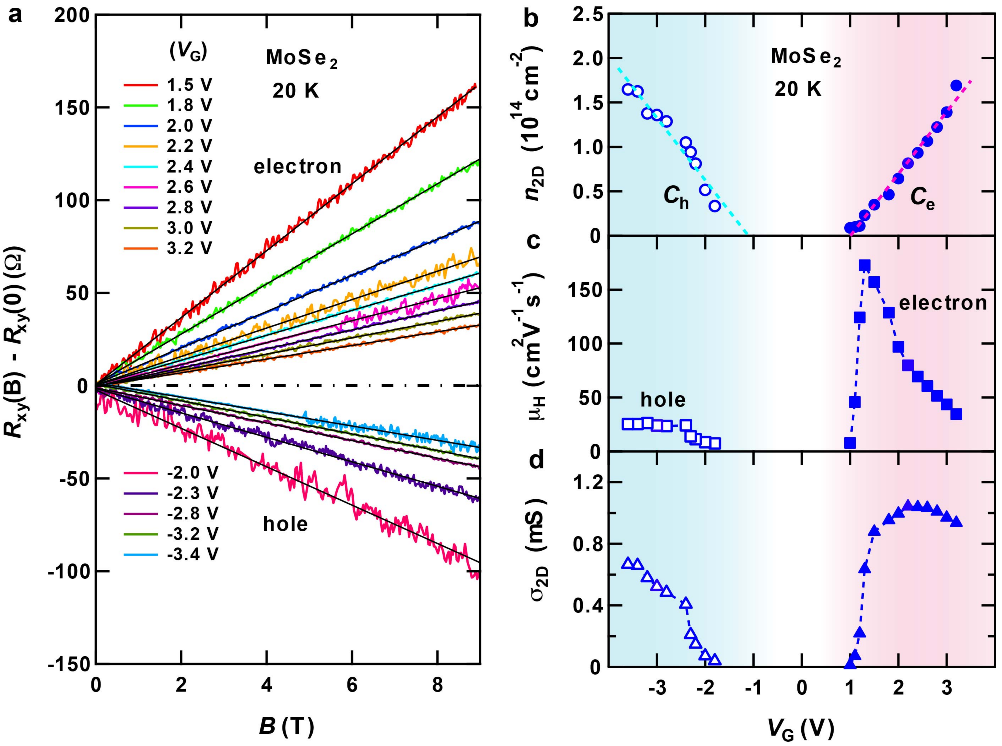

Supplementary Figure S2 | Hall effect measurements and tranport properties of a $MoSe_2$ thin flak EDLT device at 20 K. (a) Hall resistances $[R_{xy} - R_{xy}(0)]$ at different V_G are poltted as a function of magetic field B. The slopes of the linear fittings (blak solid lines) change sign when V_G switches from positive to negative, indicating the change of carrier polarity with gate voltages. (b) Sheet carrier density n_{2D} determined from the Hall effect, (c) Hall mobility μ_H and (d) sheet conductivity σ_s for the same $MoSe_2$ EDLT device at 20 K plotted as a function of gate voltage. The linear dependence (dashed lines) between the carrier density and the gate voltage in (b) indicates the contribution of electrostatic processes to both electron and hole accumulation.

3. Electron transport of IL-gated Mo_2 thin flakes

We measured the transport properties of MoX_2 thin-flake EDLTs down to 2 K by varying V_G in the positive direction to access electron transport in the range in which

superconductivity has previously been observed in one member of this series, MoS_2[11]. The results are plotted as the channel sheet resistance R_s versus T in Fig. S3. Note that the data for MoS_2 that are presented in Fig. S3a were obtained from ref. 23 for comparison. All three materials displayed clear insulator–metal transitions with increasing electron density under higher V_G. Gate-induced superconductivity emerged in $MoSe_2$ at V_G = 2.4 V and developed further with the further increase in V_G, as shown in Fig. S3b. However, no superconducting transition was observed in $MoTe_2$ up to V_G = 2.5 V, at which voltage a conductivity maximum was reached. At higher V_G values, the carrier density was saturated (see Fig. S6b) and the mobility decreased, precluding the formation of a more pronounced conducting state.

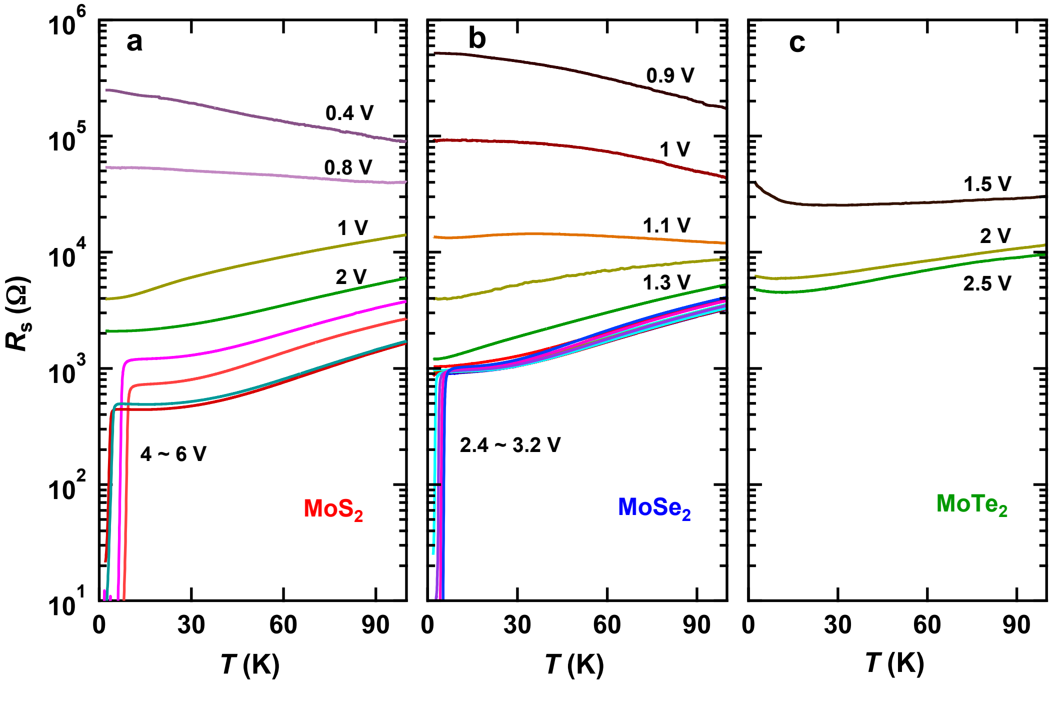

Figure S3 | Transport and superconductivity of electron-doped MoX_2 under electrostatic IL gating. Temperature dependence of the channel sheet resistance R_s at various liquid gate voltages for (a) MoS_2, (b) $MoSe_2$ and (c) $MoTe_2$ EDLTs, all using DEME-TFSI. The transport data for MoS_2 that are presented in (a) were obtained from

ref. 23 for comparison. The transport data for $MoSe_2$ that are presented in (b) were measured from the same device as that represented in Fig. 3.

4. Hole transport of IL-gated MoX_2 thin flakes

Transport measurements were also performed for the hole side for all MoX_2 devices, and the results are presented in Fig. S4. Similar insulator–metal transitions as those observed for the electron side were observed as V_G was increased in the negative direction. No superconducting transition was observed down to 2 K, most likely as a result of the low mobility and poor metallicity for hole conduction at low temperatures. However, even if hole superconductivity had been present, the critical temperature would have been lower than 2 K, which was the lowest limit attainable in this study. The attempt to measure these properties at even lower temperatures will be an interesting challenge in the near future.

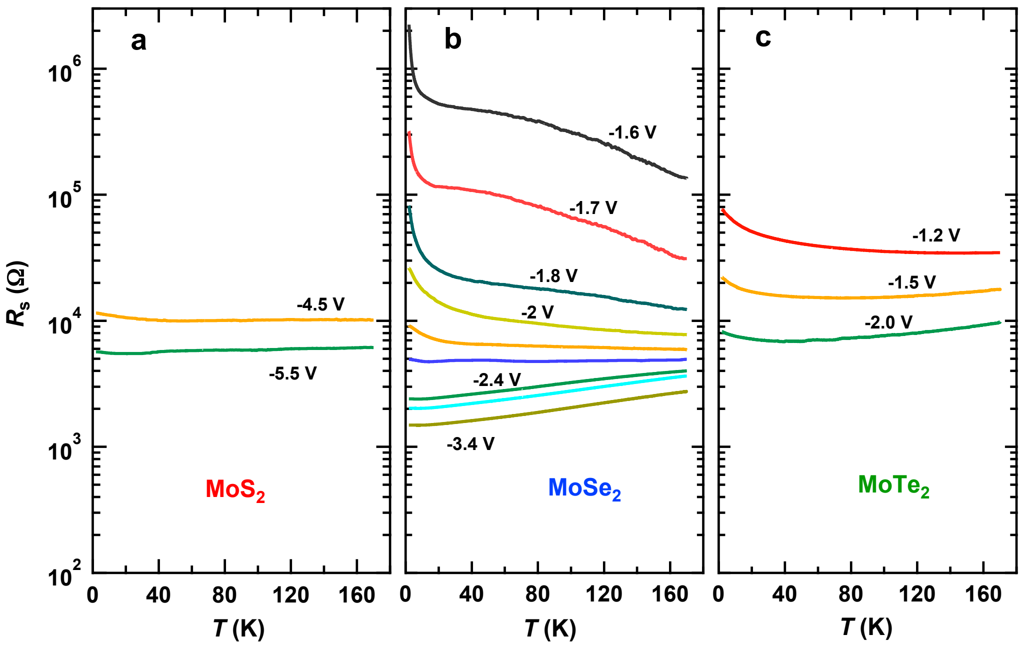

Supplementary Figure S4 | Transport properties of MoX_2 thin-flake EDLTs in the hole-doped case. Temperature dependence of the channel sheet resistance R_s at various values of V_G for (**a**) MoS_2, (**b**) $MoSe_2$ and (**c**) $MoTe_2$. The ionic liquid was DEME-TFSI. Insulator–metal transitions could be clearly observed in all three materials when the gate voltages were increased in the negative direction, but no superconducting transition was observed down to 2 K.

5. H_{c2} phase diagram of IL-gated $MoSe_2$

To further investigate the superconducting nature of IL-gated $MoSe_2$, we performed scans with magnetic fields of up to 9 T applied perpendicularly to the flake and measured the magnetoresistances for various temperatures from 2 K to 20 K. The original data acquired at V_G = 3.2 V are presented in Fig. S5a. H_{c2} is defined as the magnetic field required to recover 50% of the normal sheet resistance at 15 K. Figure S5b shows the temperature dependence of H_{c2} for $MoSe_2$ at V_G = 3.2 V and 3 V. It is evident that a higher V_G corresponded to a higher H_{c2} within the measured range of V_G, suggesting that a more robust superconducting state was formed. Because there was a linear dependence between the sheet carrier density n_{2D} and V_G, we plotted the H_{c2} versus n_{2D} phase diagram for $MoSe_2$, as shown in Fig. S5c. The MoS_2 phase diagram (red shade) was taken from ref. 23 for comparison. Both exhibited similar dome-like shapes to those observed in the phase diagram of T_c versus carrier density (Fig. 3c).

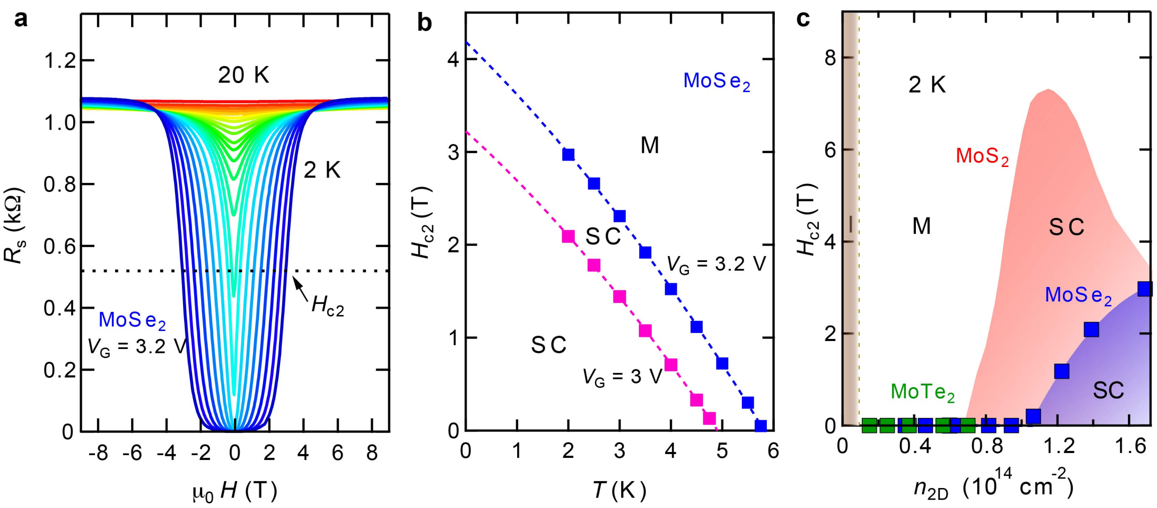

Supplementary Figure S5 | Magnetoresistances and H_{c2} phase diagrams of electron-doped $MoSe_2$ under IL gating. (**a**) Channel sheet resistance as a function of the magnetic field H for various temperatures from 2 K to 20 K at V_G = 3.2 V. The IL was DEME-TFSI. H_{c2} is defined as the magnetic field required to recover 50% of the normal sheet resistance at 15 K, as indicated by the arrow. (**b**) H_{c2} of $MoSe_2$ at T = 2 K as a function of temperature at two different liquid gate voltages, V_G = 3.2 V and V_G = 3 V. (**c**) H_{c2} of MoS_2 and $MoSe_2$ at T = 2 K as a function of carrier density. The MoS_2 phase diagrm in (c) was taken from ref. 23 for comparsion. MoS_2 and $MoSe_2$ show very similar phase diagram of superconductivity while $MoTe_2$ shows no evidence of superconductivity down to 2 K due to the insufficient maxium carrier density.

6. IL dependence and saturation behavior of carrier density in $MoTe_2$ EDLTs

As discussed in the main text, the transistor performance was related to the energy-level alignment at the interface between the IL and the MoX_2. Consequently, $MoTe_2$ exhibited the largest threshold voltage V_{th_e} for electron accumulation compared with MoS_2 and $MoSe_2$ for the same IL, DEME-TFSI. By exploiting the same mechanism, we

can further reduce V_{th_e} by replacing DEME-TFSI with other ILs with smaller work functions. Figure S6a presents the transfer curves of a $MoTe_2$ EDLT device measured using two different ILs, DEME-TFSI and BMIM-BF_4. BMIM-BF_4 has a smaller work function (3.85 eV) than DEME-TFSI (4.27 eV), as determined via ultraviolet photoemission spectroscopy measurements. Good ambipolar operation was observed in both cases. V_{th_e} shifted in the negative direction from DEME-TFSI to BMIM-BF_4, as expected, indicating a decrease in the electron injection barrier. This was confirmed by the sheet carrier densities determined from the Hall effect measurements, as shown in Figure S6b. Compared with the case of DEME-TFSI, electron accumulation began at a lower V_G and reached a higher maximum carrier density of 0.45 x 10^{14} cm^{-2} when BMIM-BF_4 was used. However, in both cases, the device exhibited a saturation of the carrier density with increasing V_G, which limited the maximum attainable carrier density and hence served as a barrier to the achievement of superconductivity in $MoTe_2$. This feature of carrier-density saturation, which was associated with a peak in the source–drain current at high V_G values of greater than 2.5 V (as seen in Fig. 4a), was confirmed in multiple devices, implying that the behavior was not related to device degradation. Such behavior has also been observed in ZnO EDLTs, in which case, significant electron tunneling between the semiconductor conduction bands and the LUMO level of the IL at large V_G values was regarded as the explanation for the carrier saturation[12]. This explanation may also be applied to the MoX_2 case because the band alignment at the interface is similar. As seen in Fig. S6b, the EDL capacitances were found to be C_e = 2.81 $\mu F/cm^2$ and 3.76 $\mu F/cm^2$ for DEME-TFSI and BMIM-BF_4, respectively. The difference

in capacitance between the two ILs was small, and therefore, the modulation of the maximum carrier density was not sufficient to induce superconductivity in $MoTe_2$.

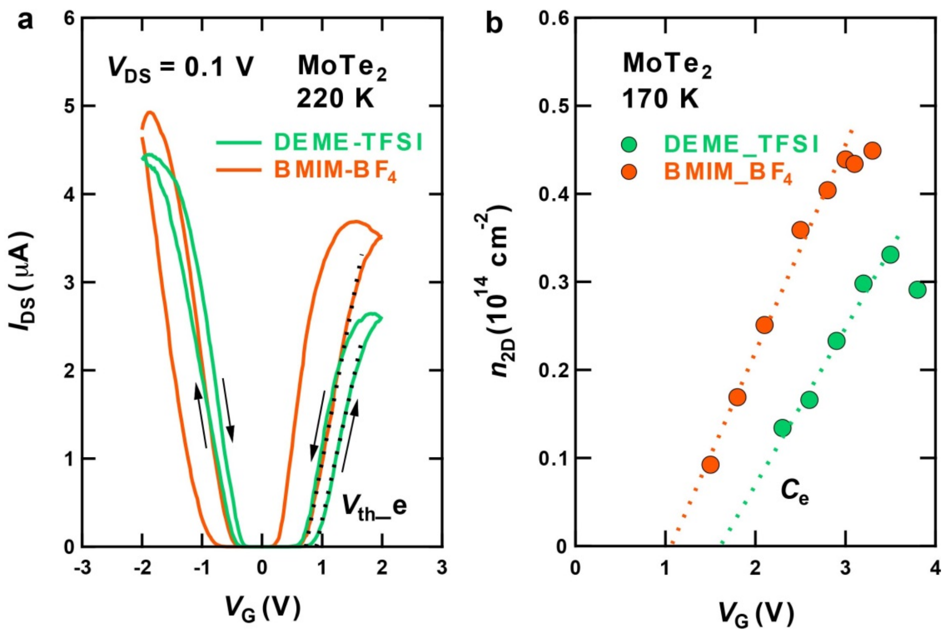

Supplementary Figure S6 | IL dependence of $MoTe_2$ thin-flake EDLTs. (a) Comparison of transfer curves of an $MoTe_2$ EDLT device with two different ILs, DEME-TFSI and BMIM-BF_4. The source–drain current I_{DS} was measured with V_{DS} = 0.1 V at 220 K. V_G was swept at a constant rate of 20 mV/s. A negative shift in the threshold voltages V_{th}_e was clearly observed when DEME-TFSI was replaced with BMIM-BF_4. (b) Sheet carrier density measured at 170 K plotted as a function of the gate voltage V_G for the same $MoTe_2$ EDLT device with the two different ILs.

7. Crossover from electrostatic to electrochemical doping in electrolyte-gated WS_2

In order to achieve a higher carrier density beyond the electrostatic limit, we used the $KClO_4$/PEG electrolyte as ionic media and carried out systematic gating experiments up

to 12 V for a WS_2 device. Figure S7 presents the channel current I_{DS} and leak current I_G as a function of V_G measured at 300 K. We can see there were three distinct regions for I_G in response to the increase of V_G. The first increasing regime ($0 < V_G < \sim 3$ V) was contributed from the electrostatic electron accumulation, so we called it an electrostatic regime, where I_G was negligible and the hysteresis of $I_{DS} - V_G$ curve was small. The second upturn of I_{DS} ($V_G > \sim 7$ V) was attributed to electrochemical doping. This was supported by the large hysteresis that were required to distract the intercalated ions from the flake as V_G was decreased to zero and a much higher carrier density confirmed by Hall effect measurement (see Fig. 5a). In the intermediate regime ($\sim 3 < V_G < \sim 7$ V) between the electrostatic and electrochemical regimes, I_{DS} nearly kept unchanged but I_G started to increase, which may imply the occurrence of PEG electrolysis or weak electrochemical doping. Since the device can be mostly recovered from the chemical doped states by decreasing V_G, we can realize a crossover from electrostatic to electrochemical doping and control the doping level by changing V_G in a reversible way. It is noted that the critical V_G for each regime is strongly dependent on materials, ionic media, device configuration, scan rate of V_G and also the waiting time at a fixed V_G. For example, a dramatic increase of I_{DS} would be observed if we keep waiting at $V_G = 6$ V for a long time even it locates in the intermediate regime.

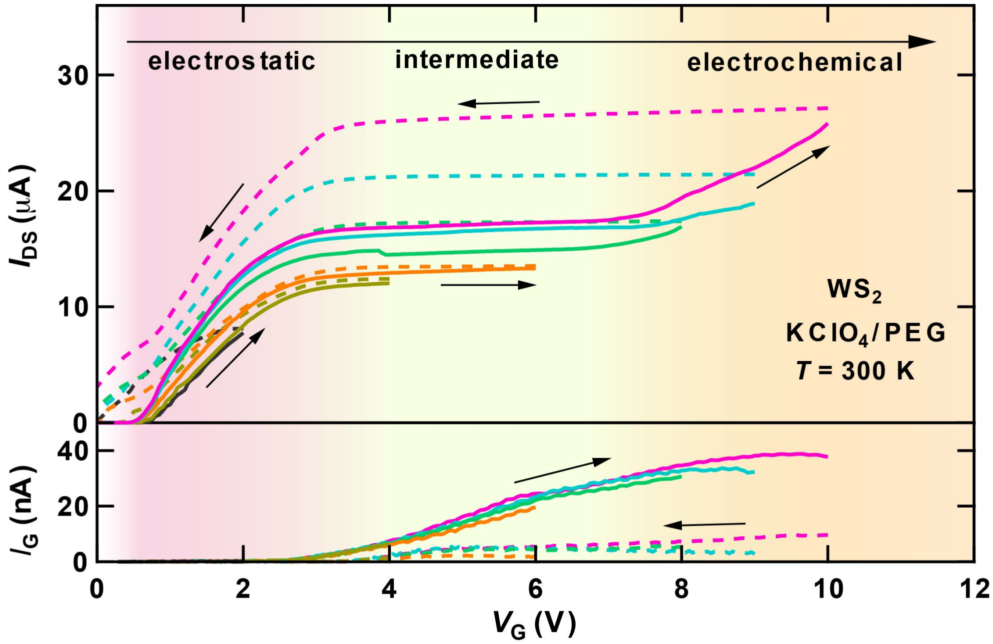

Supplementary Figure S7 | I_{DS} and I_G – V_G characteristics in electron-doped WS_2 with $KClO_4$/PEG as ionic media. Channel current I_{DS} and leak current I_G versus V_G characteristics of a WS_2 device obtained by sweeping the $KClO_4$/PEG gate at 300 K at constant rate of 20 mV/s with V_{DS} = 0.1 V. The solid lines indicate the forward V_G scan of I_{DS}, followed by immediate cooling of the device for low temperature transport measurements fixed at each maximum V_G. The dashed lines were obtained via a backward scan of V_G after the device was warmed up to 300 K.

8. Hall effect measurements of electrolyte-gated WS_2

We used the Hall carrier density n_{Hall} to quantify the doping level of the flake. Figure S8 shows the Hall resistance R_{xy} as a function of magnetic field measured at 200 K for different gate voltages from the same electrolyte-gated WS_2 thin flake device as Fig. 5. The obtained values of n_{Hall} are shown in Fig. 5a. Here, n_{Hall} corresponds to the sheet carrier density n_{2D} in the electrostatic regime, whereas beyond the electrostatic regime,

n_{Hall} corresponds to the projected carrier density, which is essentially the bulk density n_{3D} multiplied by the thickness of the doped sample.

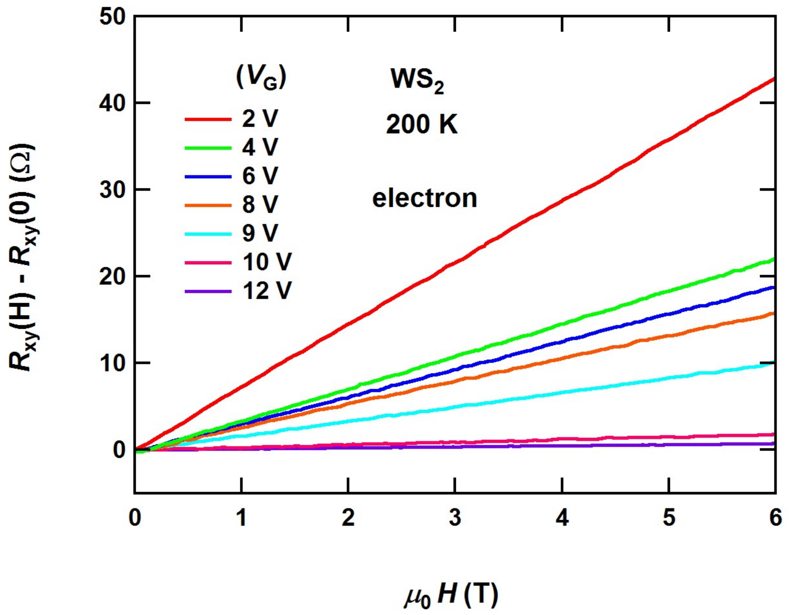

Supplementary Figure S8 | Hall effect measurements of a WS_2 thin flak device at 200 K with $KClO_4$/PEG as ionic media. Hall resistances [R_{xy} – $R_{xy}(0)$] at different V_G are poltted as a function of magetic field H.

References:

1. Sugai, S. & Ueda, T. High-pressure Raman spectroscopy in the layered materials 2*H*-MoS_2, 2*H*-$MoSe_2$, and 2*H*-$MoTe_2$. *Phys. Rev. B* **26**, 6554-6558 (1982).
2. Balendhran, S., *et al.* Two-dimensional molybdenum trioxide and dichalcogenides. *Adv. Funct. Mater.* **23**, 3952-3970 (2013).
3. Somoano, R. B., Hadek, V., Rembaum, A., Samson, S. & Woollam, J. A. Alkaline-earth intercalates of molybdenum-disulfide. *J. Chem. Phys.* **62**, 1068 (1975).

4. Schutte, W. J., Boer, J. L. d. & Jellinek, F. Crystal structures of tungsten disulfide and diselenide. *J. Solid State Chem.* **70**, 207-209 (1987).

5. Kumar, A. & Ahluwalia, P. K. Electronic structure of transition metal dichalcogenides monolayers 1*H*-MX_2 (M = Mo, W; X = S, Se, Te) from ab-initio theory: new direct band gap semiconductors. *Eur. Phys. J. B* **85**, 186 (2012).

6. Shimada, T., Ohuchi, F. S. & Parkinson, B. A. Work function and photothreshold of layered metal dichalcogenides. *Jpn. J. Appl. Phys.* **33**, 2696-2698 (1994).

7. Britnell, L., *et al.* Strong light-matter interactions in heterostructures of atomically thin films. *Science* **340**, 1311-1314 (2013).

8. Ueno, K., *et al.* Discovery of superconductivity in $KTaO_3$ by electrostatic carrier doping. *Nature Nanotech.* **6**, 408 (2011).

9. Xia, Y., Xie, W., Ruden, P. P. & Frisbie, C. D. Carrier localization on surfaces of organic semiconductors gated with electrolytes. *Phys. Rev. Lett.* **105**, 036802 (2010).

10. Xie, W. & Frisbie, C. D. Organic electrical double layer transistors based on rubrene single crystals: examining transport at high surface charge densities above 10^{13} cm^{-2}. *J. Phys. Chem. C* **115**, 14360-14368 (2011).

11. Ye, J. T., *et al.* Superconducting dome in a gate-tuned band insulator. *Science* **338**, 1193-1196 (2012).
